# Enhanced Interferon-β Response Contributes to Eosinophilic Chronic Rhinosinusitis

**DOI:** 10.3389/fimmu.2018.02330

**Published:** 2018-10-16

**Authors:** Yong Ju Jang, Ji Youn Lim, Seoyeon Kim, Yoo La Lee, Mi-Na Kweon, Ji Heui Kim

**Affiliations:** ^1^Department of Otorhinolaryngology-Head and Neck Surgery, Asan Medical Center, University of Ulsan College of Medicine, Seoul, South Korea; ^2^Department of Convergence Medicine, Asan Medical Center, University of Ulsan College of Medicine, Seoul, South Korea

**Keywords:** chronic rhinosinusitis, nasal polyposis, IFN-β, eosinophil, type 2 immune response, CCL11

## Abstract

Type I interferon (IFN-I, including IFN-α and IFN-β) response has been implicated in eosinophilic inflammation, in addition to antiviral function. This study aimed to investigate the role of IFN-I in the pathogenesis of eosinophilic chronic rhinosinusitis (ECRS). IFN-α, IFN-β, cytokine expression, and IFN-β cellular localization in the sinonasal tissue from control subjects and ECRS patients with nasal polyps (NP) were determined using real time-PCR, ELISA, and immunohistochemistry. ECRS was induced in wild-type (WT) and IFNAR1 knockout (*Ifnar1*^−/−^) mice by intranasal challenge with *Aspergillus* protease and ovalbumin. Stromal cells cultured from NP tissue were stimulated by exogenous IFN-β, and their CCL11 production and IRF3, IRF7, STAT1, STAT2, and IRF9 gene and/or protein expression were measured. IFN-β, IL-5, IL-13, and CCL11 expression was higher in the NP tissue from ECRS patients, compared to the control group. IFN-β was highly colocalized with the CD11c+ cells in NP. IFN-β levels positively correlated with IL-5, IL-13, and CCL11 levels as well as the number of eosinophils in the NP tissue and CT score. The histological severity of ECRS, levels of IL-4, IL-5, IL-13, and CCL11 in the nasal lavage fluid, and total serum IgE levels were less in *Ifnar1*^−/−^ mice than in WT mice. CCL11 production, and STAT1 and STAT2 mRNA and STAT1, phospho-STAT1, and phospho-STAT2 protein expression were significantly increased by exogenous IFN-β in NP stromal cells. Our data suggest that IFN-β response was upregulated in ECRS and may play role in ECRS development. IFN-β may contribute to ECRS by enhancing CCL11 production. Thus, increased IFN-β response in the sinonasal mucosa may underlie ECRS pathogenesis.

## Introduction

Eosinophilic chronic rhinosinusitis (ECRS) is an inflammatory disease characterized by remarkable infiltration of eosinophils and elevated type 2 response, and is frequently associated with nasal polyp (NP) tissue (1). ECRS tends to be more severe than non-ECRS, and recurrence is very common despite surgical and clinical therapy (2–4). Therefore, a number of studies have been conducted to find therapeutic targets for ECRS.

Type I interferons (IFN-I), namely, IFN-α and IFN-β, are principal cytokines involved in the innate immunity against viruses or tumors. IFN-I bind a heterodimeric transmembrane receptor called the IFNα receptor (IFNAR) composed of IFNAR1 and IFNAR2 subunits. IFN-I potently modulate both innate and adaptive immune responses, promoting antigen presentation, natural killer cell functions, and development of effector T and B cell responses, while controlling pro-inflammatory pathways and production of other cytokines (5, 6). Among the various roles of IFN-I, the role of IFN-I response in the imbalance between type 1 and 2 immune responses has been actively explored, but continues to remain unresolved. Some studies showed that IFN-I is a potent stimulator of type 1 response and suppressor of type 2 response (7, 8), while other studies showed that the shift from type 1 to type 2 immune response is caused by IFN-β in patients with multiple sclerosis, and that the adherence of eosinophils to endothelial cells and survival of eosinophils is promoted by IFN-I (9–12). Zhang et al. showed a potential association between IFN-I and type 2 immune response-skewed eosinophilic inflammation in patients with CRS (13). However, they could not clearly explain the role of IFN-I response in ECRS.

In this study, we aimed to investigate the role of IFN-I in ECRS pathogenesis by investigating the expression of IFN-I, type 2 cytokines, and chemokines in the NP tissues from non-ECRS and ECRS patients. ECRS was induced in wild-type (WT) and IFNAR1 knockout (*Ifnar1*^−/−^) mice, to compare the phenotype as well as cytokine production in both groups, to further elucidate the role of IFN-I in ECRS pathogenesis.

## Materials and methods

### Study subjects

Thirty-one adult patients with CRS with NP (CRSwNP) were recruited from the Department of Otorhinolaryngology-Head and Neck Surgery, Asan Medical Center, between December 2014 and May 2016. CRSwNP was diagnosed based on the established criterion for CRSwNP, defined by the European Position Paper on Rhinosinusitis and Nasal Polyps (2012) guidelines based on the history, nasal endoscopy, and computed tomography (CT) of the paranasal sinuses (14). Subjects with antrochoanal polyps, fungal sinusitis, classic allergic fungal sinusitis, aspirin-exacerbated respiratory disease, immunodeficiency, pregnancy, coagulation disorder, cystic fibrosis, or primary ciliary dyskinesia were excluded. Patients who had received decongestants, antibiotics, and topical and/or systemic corticosteroids for a minimum of 4 weeks prior to the surgery were excluded. No patient had acute respiratory infection. NP tissues were harvested during routine functional endoscopic sinus surgery. ECRS with NP was defined as the proportion of eosinophils exceeding 10% of total inflammatory cells (15, 16). Control tissues were collected from the uncinate process of 7 patients undergoing septoplasty to correct nasal septal deviation, without any other inflammatory nasal diseases such as allergic rhinitis or sinusitis.

Atopic status was evaluated using the skin prick test (Allergopharma; Hermann-Körner-Straße, Reinbeck, Germany) or AdvanSure™ AlloStation (LG Life Science, Seoul, Korea) by detecting specific IgE antibodies to various common inhalant allergens. Asthma and aspirin intolerance were diagnosed based on clinical history and tests such as pulmonary function test and challenge test. Preoperative Sino-nasal outcome test (SNOT-22) score, polyp grading according to the Davos classification, Lund-Mackay CT scores, CT score ratios for the ethmoid sinus and maxillary sinus (E/M ratio), and blood eosinophil counts were obtained (17, 18). Tissues from the NP and control groups were used for quantitative real-time PCR, ELISA, and histological analysis. NP tissues were also used for stromal cell culture. This study was conducted in accordance with the Declaration of Helsinki and approved by the Institutional Review Board of Asan Medical Center (2014-1199) and all participants provided written informed consent. Clinical and demographic findings of patients are summarized in Table 1.

**Table 1:** Clinical characteristics of control subjects and patients with CRS with NPs.

**Characteristics**	**Control (*n* = 7)**	**Non-eosinophilic CRSwNP (*n* = 12)**	**Eosinophilic CRSwNP (*n* = 19)**
Age (y), median (range)	41 (28–56)	47 (32–76)	52 (30–71)
Gender (M:F)	6:1	8:4	13:6
Atopy, number (%)	0 (0)	4 (33.3)	11 (57.9)^*^
Asthma, number (%)	0 (0)	1 (8.3)	3 (15.8)
Aspirin intolerance, number (%)	0 (0)	0 (0)	0 (0)
SNOT-22 score, median (range)	14 (8–32)	40 (16–79)	37 (9–85)
Polyp grade, median (range)	0	3 (3–6)	4 (2–6)
Lund-Mackay CT score, mean ± SD	0	13.4 ± 3.6	17.0 ± 3.4^†^
E/M ratio, mean ± SD	0	1.6 ± 0.7	2.3 ± 0.8^†^
Blood eosinophil (cells/μL), median (range)	140 (63–200)	200 (2–462)	279 (89–757)^‡^
Methodology used, number			
Real-time PCR	7	12	19
Tissue homogenate ELISA	7	12	19
Tissue immunohistochemistry	3	12	19

* *P < 0.01, eosinophilic CRSwNP vs. non-eosinophilic CRSwNP, chi-square test*.

† *P < 0.01, eosinophilic CRSwNP vs. non-eosinophilic CRSwNP, Mann-Whitney U test*.

‡ *P < 0.01, eosinophilic CRSwNP vs. controls, Mann-Whitney U test*.

*SNOT, Sino-nasal outcome test*.

### Real-time PCR for Type I IFN in human tissue

The expression levels of *IFNA* family genes and *IFNB* were evaluated using quantitative real-time PCR. Detailed procedures are described in the online supplement.

### ELISA for cytokines and chemokines in the human tissue homogenates

IFN-α, CXCL-10/IP-10, and IL-8 levels were measured using a multiplex immunoassay (ProcartaPlex® Multiplex Immunoassays; eBioscience, Vienna, Austria). IL-4, IL-5, and IL-13 levels were measured with the Human Magnetic Luminex Screening Assay (R&D Systems, Minneapolis, MN), and IFN-β and CCL11 levels, with DuoSet Human ELISA Kit (R&D Systems), according to the manufacturers' instructions. Detailed procedures are described in the online supplement.

### Immunohistochemistry

NP and control tissues obtained during surgery were fixed with formalin and embedded in paraffin, and sections were immunohistochemically stained for IFN-β by using a BenchMark XT automatic immunostaining device (Ventana Medical Systems, Tucson, AZ) with the ultraView Universal DAB Detection Kit (Ventana Medical Systems) according to the manufacturer's instructions. Four-micrometer-thick sections were transferred onto silanized/charged slides and allowed to dry for 10 min at room temperature, followed by 20 min in an incubator at 65°C. Sections were made by Protease 1 (Ventana Medical Systems) for 1 min and incubated with polyclonal rabbit anti-human IFN-β (1:1,000, Millipore, Billerica, MA) for 16 min in the autoimmunostainer. In control experiments sections were incubated with the same concentrations of control rabbit IgG (Millipore). IFN-β^+^ cells observed under the high-power field (HPF, ×400 magnification) were enumerated by a blinded pathologist unaware of the experimental groups, and 6–8 fields were randomly selected and analyzed (13).

For immunofluorescence assay, rehydrated sections were blocked with 3% goat serum/0.3% Tween20/phosphate-buffered saline (PBS), followed by incubation with rabbit anti-human IFN-β (1:250; Millipore) and mouse anti-human CD11c (1:100; Novus Biologicals, Littleton, CO) antibodies overnight at 4°C. In control experiments sections were incubated with the same concentrations of control rabbit IgG (Millipore) and control mouse IgG (Millipore). Sections were washed three times with PBS, and incubated with secondary antibody donkey anti-rabbit IgG Alexa Fluor® 594 (1:200; Abcam, Cambridge, UK) and donkey anti-mouse IgG-Alexa® 488 (1:200; Abcam) for 1 h at room temperature in the dark. For double staining of IFN-β and blood dendritic cell antigen 2 (BDCA2, CD303), rabbit anti-human IFN-β (1:250; Millipore) and goat anti-human BDCA2 (1:20; Novus Biologicals) were used as the primary antibody, respectively. Control sections were incubated with the same concentrations of control rabbit IgG (Millipore) and control goat IgG (R&D Systems). Donkey anti-rabbit IgG FITC (1:100; Santa Cruz Biotechnology, Santa Cruz, CA) and donkey anti-goat IgG CFL594 (1:100; Santa Cruz Biotechnology) were used as the secondary antibody, respectively. The sections were washed three times with PBS and mounted with Vectashield mounting medium containing 4,6-diamidino-2-phenylindole (DAPI; Vector Labs, Burlingame, CA) and examined using a confocal laser scanning microscope (ZEISS LSM 780; Carl Zeiss Microscopy GmbH, Jena, Germany).

### Histological and immunological analyses of ECRS mice

C57BL/6 mice (age, 7–8 weeks; weight, 20–25 g) were purchased from Orient Bio Inc., (Sungnam, Korea). *Ifnar1*^−/−^ mice (C57BL/6 background) were purchased from B&K Universal Ltd., (Hull, UK). ECRS was induced in 7 C57BL/6 WT and 7 *Ifnar1*^−/−^ mice, as described previously with slight modifications (19, 20). ECRS was induced by intranasal challenge in the head-down position with a mixture of 2 U protease from *Aspergillus oryzae* (Sigma-Aldrich, St. Louis, MO) and ovalbumin (OVA; Worthington Biochemical Corporation, Lakewood, NJ) diluted in sterile PBS to a total volume of 20 μL, 3 times a week for 6 weeks. The total volume of intranasally instilled fluid was 20 μL, and the mouse's head was kept down for 30 s after the challenge to prevent influx of reagents into the lung. Control mice (7 WT and 7 *Ifnar1*^−/−^) were intranasally challenged with 20 μL of PBS. All experiments were conducted according to the National Institutes of Health Guide for the Care and Use of Laboratory Animals and approved by the Institutional Animal Care and Use Committee of the Asan Institute for Life Science (2015-12-012).

Blood samples were taken via cardiac puncture under deep anesthesia. And then, nasal lavage fluid was collected as described previously (21). Briefly, a 22-gauge catheter was inserted into the tracheal opening in the direction of the posterior choanae under deep anesthesia. About 400 μL of PBS was gently introduced into the nasal cavity, and the fluid from the nostril was collected and centrifuged. Supernatants were stored at −80°C until analysis. Finally, the mice were killed and decapitated.

For histological analysis of mice sinonasal mucosa, the skulls were decalcified after fixation with 10% neutral-buffered formalin for 24 h, embedded in paraffin, coronally sectioned at a thickness of 4 μm, and stained with hematoxylin and eosin (H&E) and Sirius red for eosinophils (21, 22). The true maxillary sinus and ethmoid labyrinths were identified. The number of infiltrated eosinophils/HPF in the lamina propria from 6 randomly selected areas (3 different areas on each of right and left sides) was counted by 2 blinded examiners. To assess mucosal hypertrophy, a histological feature of sinusitis, maximal mucosal thickness was measured at the transition zone of the olfactory and respiratory epithelia by using an image analysis system (cellSens Standard 1.7; Olympus, Tokyo, Japan) (23).

IL-4, IL-13, KC/CXCL1, and MIP-2/CXCL2 concentrations in the nasal lavage fluid were measured with a multiplex immunoassay (ProcartaPlex® Multiplex Immunoassay; eBioscience) according to the manufacturer's instructions. IL-5 and CCL11 levels were measured using DuoSet Mouse ELISA Kit (R&D Systems) according to the manufacturer's instructions. Total serum IgE levels were measured with BD OptEIA™ Mouse IgE ELISA Set (BD Pharmingen, San Diego, CA).

### Assessment of IFN-β-induced CCL11 production in the NP stromal cells

NP stromal cells were isolated from the NP tissue, as described previously (24). About 1 × 10^5^ stromal cells were plated in 24-well tissue culture plates (Corning) and stimulated by 1 U/mL and 10 U/mL IFN-β (PBL, Picataway, NJ) for 48 h. The supernatants collected after 48 h of IFN-β stimulation were used to measure CCL11 concentration using the DuoSet Human ELISA Kit (R&D Systems). The expression of interferon regulatory factor 3 (*IRF3*), *IRF7*, signal transducer and activator of transcription 1 (*STAT1*), *STAT2*, and *IRF9* in NP stromal cells was assessed using quantitative real-time PCR. The PCR primer sets used for the detection of *IRF3, IRF7, STAT1, STAT2*, and *IRF9* are listed in Table E1. STAT1, STAT2, phospho-STAT1 (pSTAT1), and pSTAT2 protein expression levels were measured using Western blot analysis. Detailed methods are provided in the online supplement.

### Statistical analysis

The groups were compared using the non-parametric repeated measurement ANOVA and chi-square test and the paired comparisons were analyzed using nonparametric Friedman test. Correlations were evaluated by Spearman's correlation analysis. Data were analyzed using GraphPad Prism v.4.00 (GraphPad Software Inc.,). Statistical significance was defined as *P* < 0.05.

## Results

### IFN-β are overexpressed in the NP tissue from ECRS

**Figure 1:**
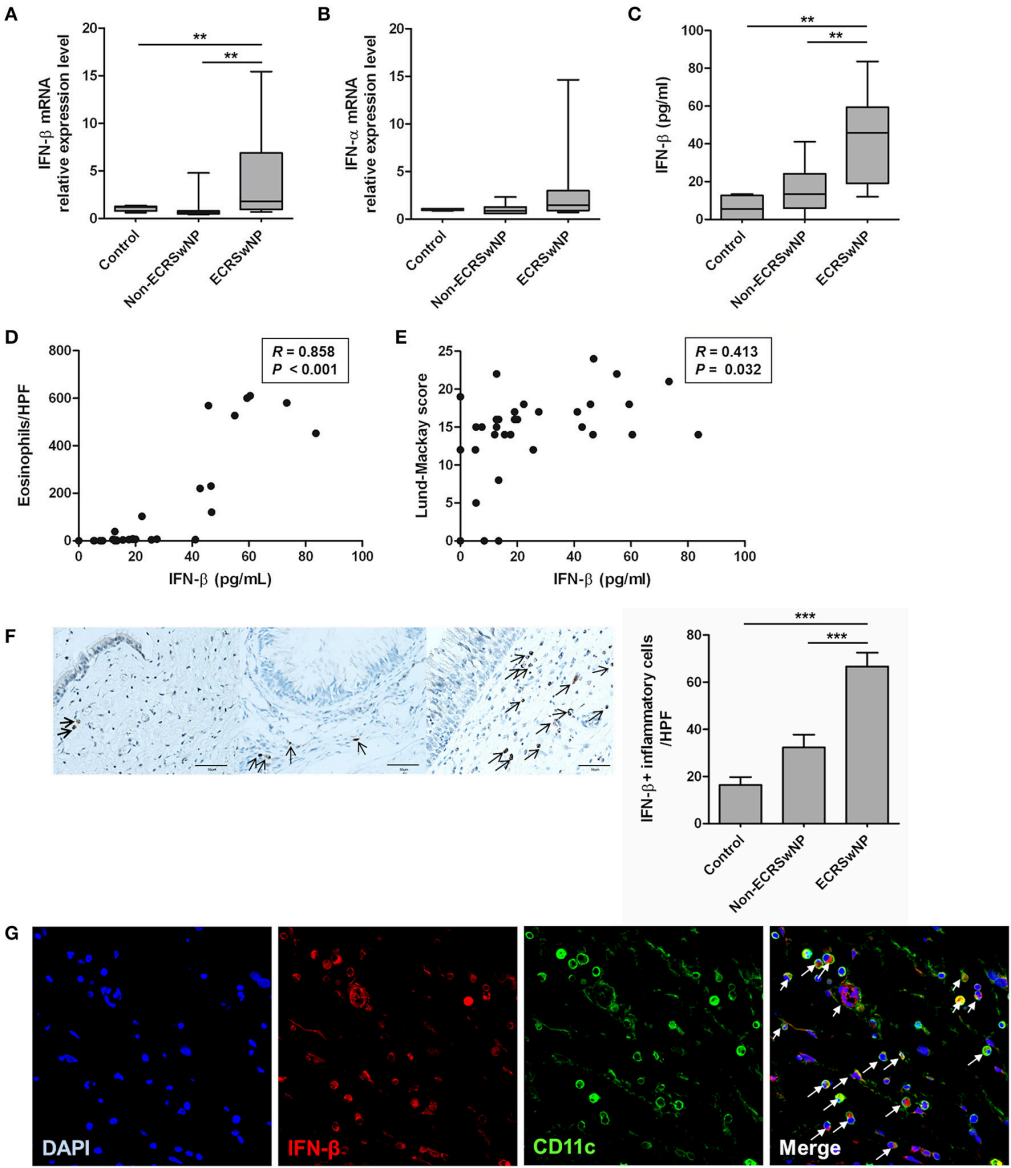
Expression of type I IFN in human sinonasal tissue. **(A,B)** IFN-β mRNA and protein levels are enhanced in tissue homogenates from eosinophilic CRS with NP (ECRSwNP) patients. **(C)** On the other hand, there are no differences in the IFN-α family mRNA expression between control, non-eosinophilic CRSwNP (non-ECRSwNP), and ECRSwNP groups. **(D,E)** IFN-β level positively correlated with the number of eosinophils in the sinonasal tissue and Lund-Mackay CT score in 31 patients with CRSwNP. **(F)** In addition, IFN-β+ cells (indicated by arrows) are more frequently detected in the lamina propria of ECRSwNP tissues compared with the controls and non-ECRSwNP (magnification, ×400; scale bar, 50 μm). Horizontal bars indicate the medians. **P < 0.01; ***P < 0.001, repeated measurement ANOVA with Tukey's multiple comparison test **(A–C,F)** and Spearman correlation test **(D,E)**. **(G)** In immunofluorescence assay for NP tissue, IFN-β (red fluorescence) was frequently detected in CD11c cells (green fluorescence). Nuclei were counterstained with 4',6-diamidino-2-phenylindole (DAPI; blue fluorescence). In the merged image, cells indicated by arrows show co-localization of IFN-β and CD11c cells. The immunofluorescence results are representative of 5 different subjects (Magnification, ×400).

We compared the expression of IFN-I (IFN-α and IFN-β) from sinonasal tissues to evaluate whether IFN-I expression in the NP tissue from the ECRS group differs from that in the NP tissue from non-ECRS group and the uncinate process mucosa from the control group. The IFN-β mRNA levels were higher in the NP tissue from the ECRS group compared to the tissues from the non-ECRS and control groups (*P* = 0.007; Figure 1A). However, mRNA levels of the IFN-α family did not significantly differ among the 3 groups (Figure 1B), and IFN-α level in all samples was undetectable by ELISA. IFN-β level measured by ELISA was also higher in the tissue from ECRS compared to the tissues from the non-ECRS and control groups (*P* = 0.001; Figure 1C). IFN-β level positively correlated with the number of eosinophils in the NP tissue (*R* = 0.858, *P* < 0.001; Figure 1D) and Lund-Mackay CT score (*R* = 0.413, *P* = 0.032; Figure 1E) in the patients with CRSwNP.

In the sinonasal mucosa, IFN-β^+^ cells were mainly localized in the lamina propria and significantly increased in the eosinophilic NP tissues compared with the control and non-eosinophilic groups (*P* < 0.001; Figure 1F). IFN-β is produced not only by innate immune cells such as dendritic cells (DCs) and macrophages (after activation of pattern-recognition receptors), but also by non-immune cells such as fibroblasts and epithelial cells (6). We focused on CD11c+ cells because CD11c is expressed on most DCs as well as monocytes and macrophages. CD11c^+^ cells are increased in the eosinophilic NP tissues compared to the non-eosinophilic NP tissues (25). Therefore, we performed double immunofluorescence staining by using anti-IFN-β and anti-CD11c antibodies. IFN-β and CD11c^+^ cells were highly colocalized in NP tissues (Figure 1G).

### Type 2 cytokines and eosinophil chemokines are positively correlated with IFN-β

**Figure 2:**
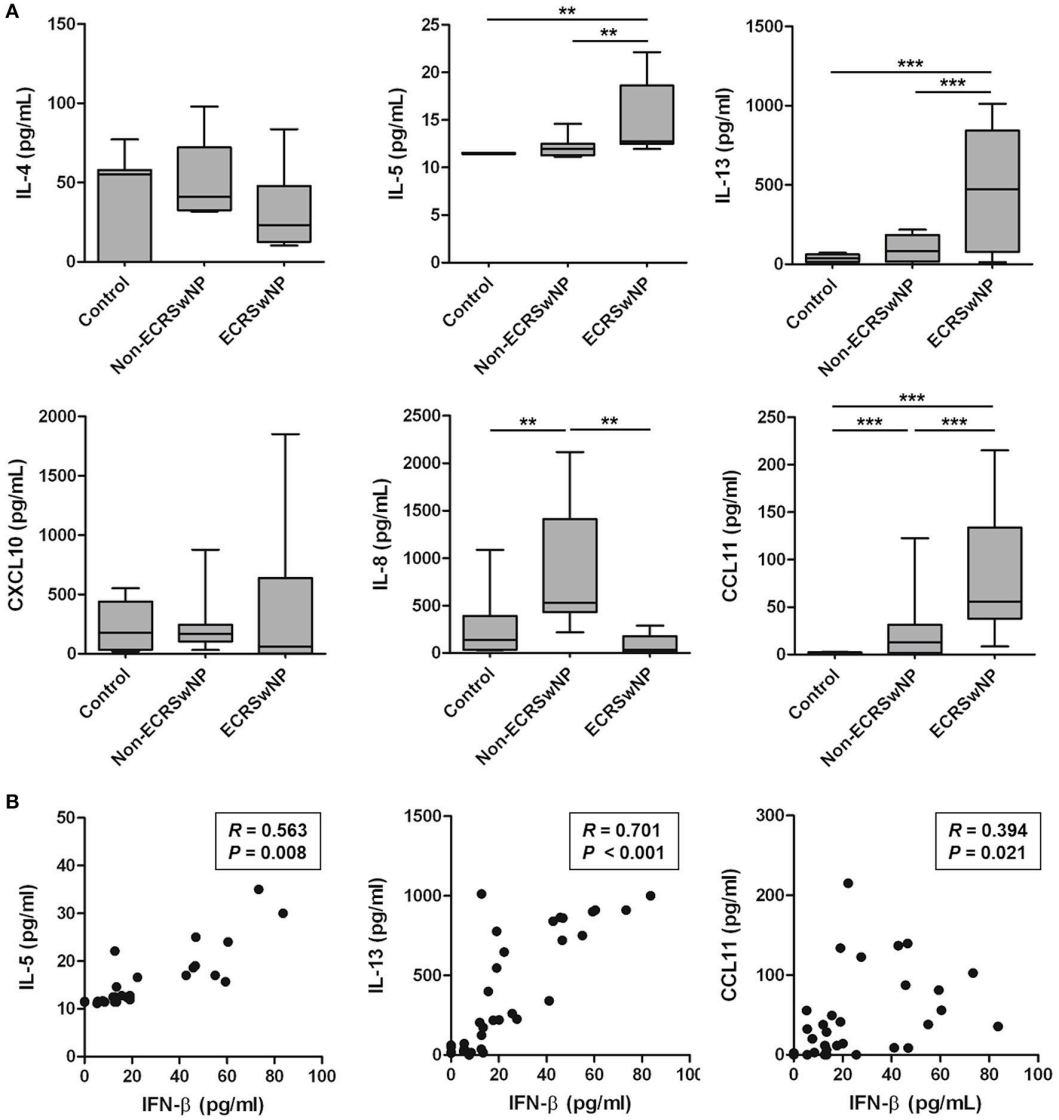
Production of type 2 cytokines and eosinophil chemokines in human sinonasal tissue homogenates. **(A)** IL-5, IL-13, and CCL11 production was increased in the tissue homogenates from eosinophilic CRS with NP (ECRSwNP). **(B)** There is a significant positive correlation of IFN-β with IL5, IL-13, or CCL11. Horizontal bars indicate the medians. **P < 0.01; ***P < 0.001, repeated measurement ANOVA with Tukey's multiple comparison test **(A)** and Spearman correlation test **(B)**.

Because eosinophilic NP tissues are characterized by type 2-skewed cytokine profile (26), we analyzed the correlation between IFN-β, which is elevated in eosinophilic NP tissue, and type 2 cytokines and chemokines in human tissue. Significant increase was noted in the levels of IL-5, IL-13, and CCL11 in the NP tissue from ECRS compared to the corresponding control and non-ECRS subjects (*P* < 0.01; Figure 2A). However, no significant differences were noted in the levels of IL-4 and CXCL10 between these 3 groups (*P* > 0.05; Figure 2A). On the other hand, the level of IL-8, a neutrophil chemokine, was higher in the NP tissue from non-ECRS subjects compared to that from the control subjects and ECRS subjects (*P* = 0.004; Figure 2A).

IFN-β level positively correlated with the levels of IL-5 (*R* = 0.563, *P* = 0.008), IL-13 (*R* = 0.701, *P* < 0.001), and CCL11 (*R* = 0.394, *P* = 0.021; Figure 2B). However, no significant correlation was noted between the levels of IFN-β and other cytokines and chemokines: IL-4 (*R* = −0.287, *P* = 0.207), CXCL10 (*R* = 0.022, *P* = 0.926), and IL-8 (*R* = −0.180, *P* = 0.435). Thus, these results suggested that IFN-β overexpression is associated with elevated type 2 response in the pathogenesis of eosinophilic NP.

### Eosinophilic sinonasal inflammation did not fully develop in *Ifnar1*^−/−^ mice

**Figure 3:**
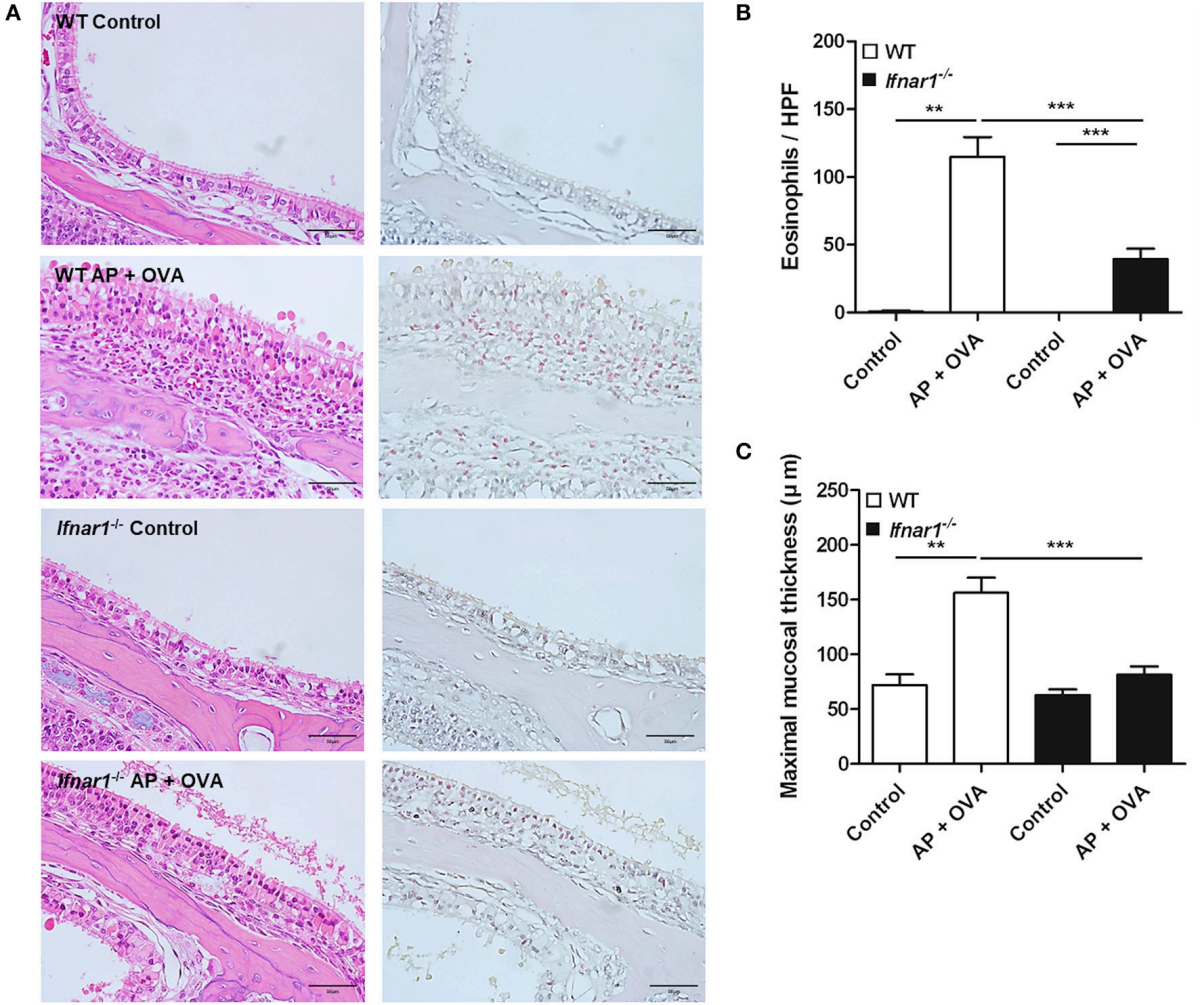
Establishment of eosinophilic chronic rhinosinusitis (ECRS) in WT and IFNAR1 knockout (*Ifnar1*^−/−^) mice. **(A)** Chronic eosinophilic inflammation in the sinonasal mucosa induced by intranasal challenge with *Aspergillus oryzae* protease (AP) and ovalbumin (OVA) was not complete in the absence of type I IFN signaling. Unlike the wild-type counterpart, epithelial hyperplasia, and inflammatory cells, in particular the eosinophils, definite infiltration was not observed in *Ifnar1*^−/−^ AP + OVA challenged mice on hematoxylin and eosin (left) and Sirius red (right) staining for eosinophil (magnification, ×400; scale bar, 50 μm). **(B,C)** Eosinophil number and maximal mucosal thickness were less in *Ifnar1*^−/−^ AP + OVA challenged mice than in WT AP + OVA challenged mice. **P < 0.01; ***P < 0.001, repeated measurement ANOVA with Tukey's multiple comparison test **(B,C)**.

**Figure 4:**
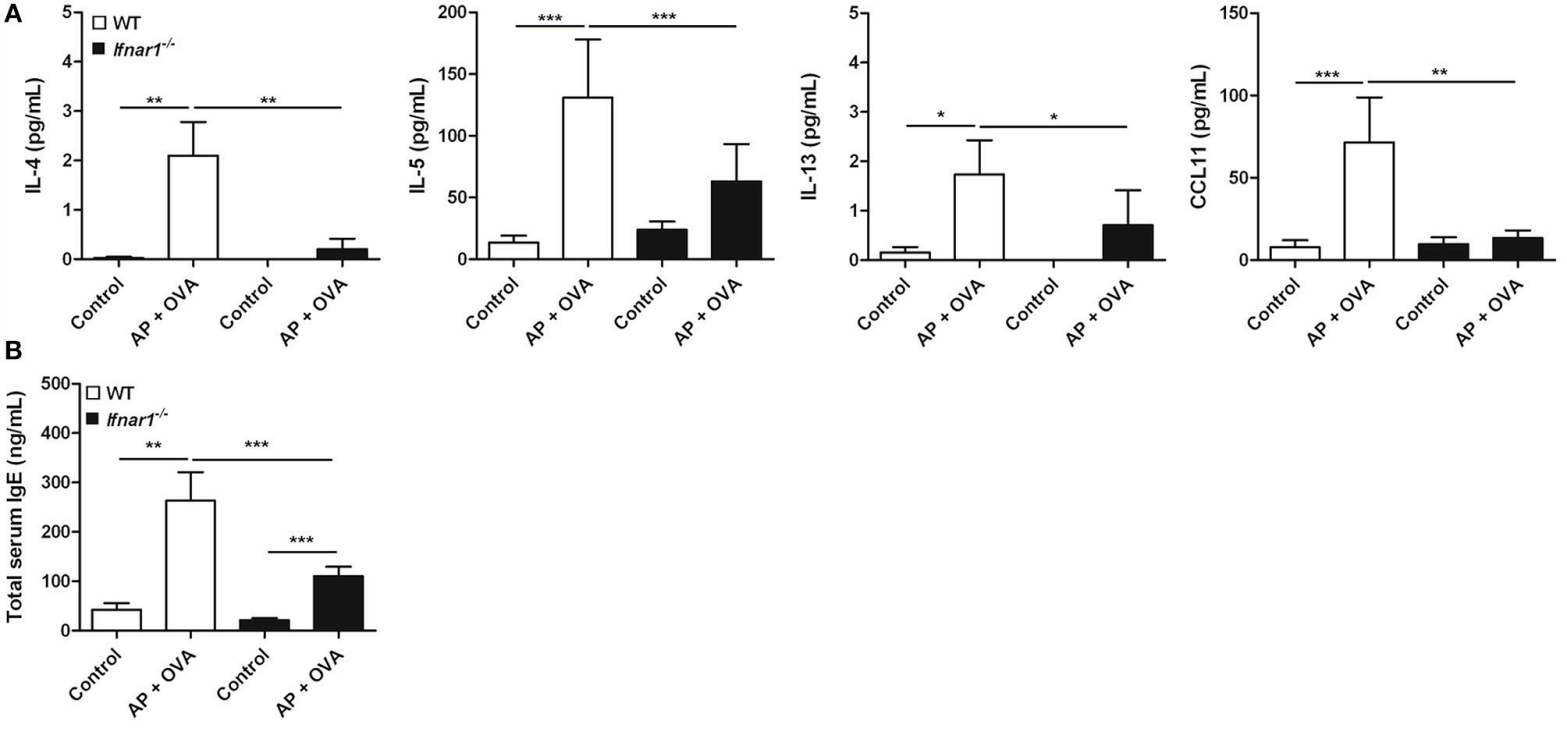
Cytokines in the nasal lavage fluid and total serum IgE levels in the murine model. **(A)** Production of type 2 cytokines such as IL-4 and IL-5, and the eosinophil chemokine CCL11 induced by *Aspergillus oryzae* protease (AP) and ovalbumin (OVA) treatment was significantly less in the *Ifnar1*^−/−^ AP + OVA challenged mice than in WT AP + OVA challenged mice. **(B)** Total serum IgE levels were also lower in the *Ifnar1*^−/−^AP + OVA challenged mice than in WT AP + OVA challenged mice. *P < 0.1; **P < 0.01; ***P < 0.001, repeated measurement ANOVA with Tukey's multiple comparison test.

To assess the role of IFN-I response in the pathogenesis of ECRS, WT and *Ifnar1*^−/−^ mice were treated with *Aspergillus oryzae* protease (AP) and OVA to develop ECRS. The severities of epithelial hyperplasia and subepithelial inflammation were less developed in *Ifnar1*^−/−^ mice than in WT (Figure 3A). The number of infiltrated eosinophils was fewer (Figure 3B) and the maximal mucosal thickness (Figure 3C) was less in *Ifnar1*^−/−^ AP + OVA challenged mice compared to WT AP + OVA challenged mice. *Ifnar1*^−/−^ mice challenged with AP + OVA 12 weeks showed less prominent eosinophilic sinusitis and formation of polypoid lesions (Figure E1) (27). The levels of IL-4, IL-5, IL-13, and CCL11 in the nasal lavage fluid were higher in the WT AP + OVA challenged mice than the *Ifnar1*^−/−^ AP + OVA challenged mice (*P* < 0.05 for each; Figure 4A). Although the serum total IgE level was higher in both WT and *Ifnar1*^−/−^ AP + OVA challenged mice compared to the corresponding control mice, it was lower in the *Ifnar1*^−/−^ AP + OVA challenged mice than the WT mice (*P* < 0.001; Figure 4B). Overall, the characteristics of ECRS were not distinctly induced in *Ifnar1*^−/−^ mice unlike in WT mice, indicating that IFN-I response may play a role in the development of chronic eosinophilic sinonasal inflammation.

### IFN-β stimulates CCL11 production in NP stromal cells

**Figure 5:**
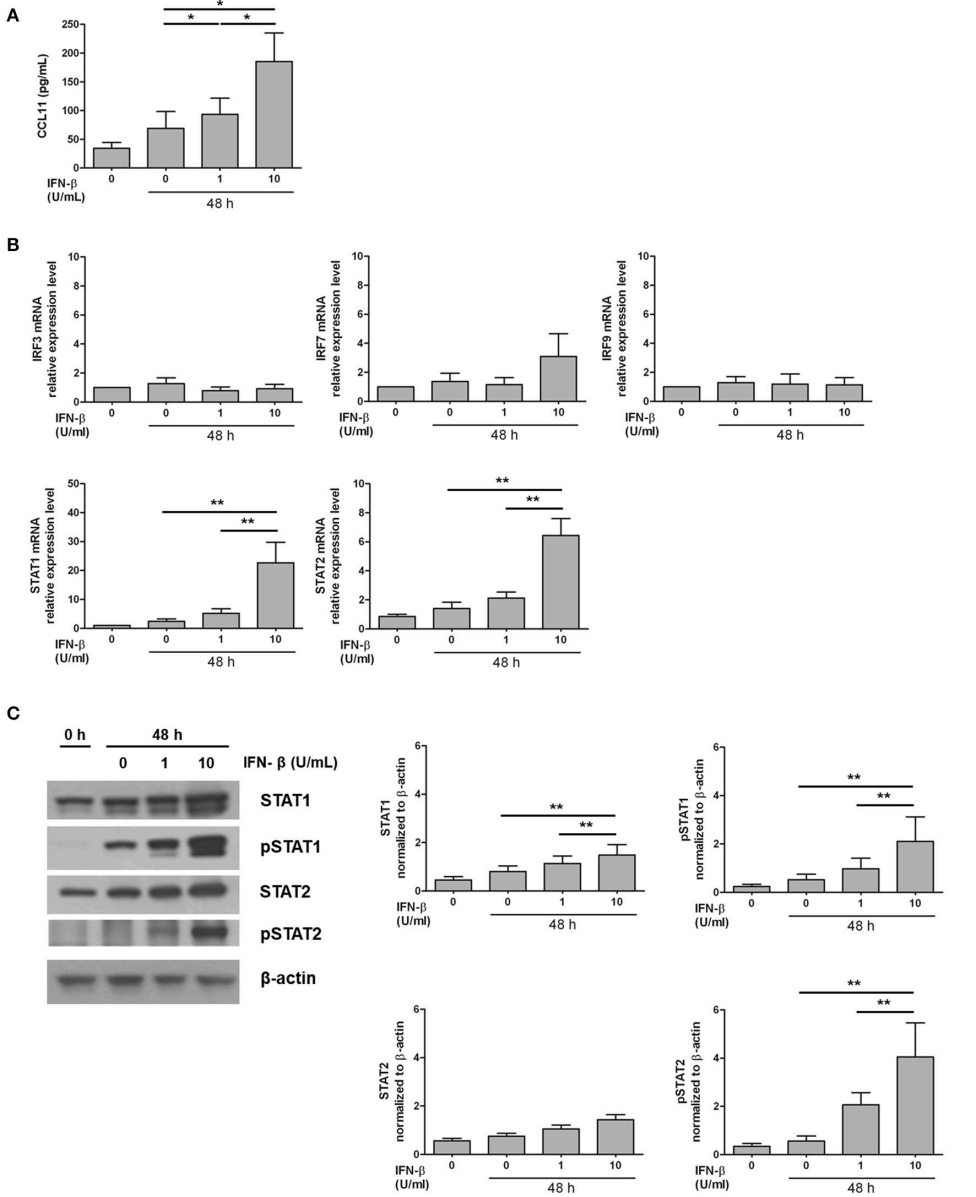
Induction of the expression of CCL11 and transcription factors by exogenous administration of IFN-β in NP stromal cell culture model. **(A)** The production of eosinophil chemokine CCL11 was increased by IFN-β stimulation in NP stromal cells in a dose-dependent manner. **(B,C)** In addition, STAT1 and STAT2 mRNA expression and STAT1, pSTAT1, and pSTAT2 protein expression were enhanced by IFN-β stimulation in NP stromal cells. *P < 0.1; **P < 0.01, repeated measurement ANOVA with the non-parametric Friedman's test.

In experiments involving both human tissue homogenates and ECRS mice, CCL11 level was distinctly increased in IFN-β-associated ECRS. Therefore, the effect of IFN-β on CCL11 production was evaluated in NP-derived stromal cells to verify the role of IFN-β in nasal tissue eosinophilia. Both 1 U/mL and 10 U/mL IFN-β significantly increased CCL11 production, with a higher increase observed with 10 U/mL (*P* < 0.05; Figure 5A).

To identify the transcription factor mediating the induction of CCL11 production by IFN-β, the expression of *IRF3, IRF7, STAT1, STAT2*, and *IRF9* was assessed. *IRF3, IRF7*, and *IRF9* expression after 48-h-long stimulation with IFN-β did not different from the group not subjected to stimulation (*P* > 0.05 Figure 5B). *STAT1* and *STAT2* expression significantly increased with increasing concentration of IFN-β (*P* < 0.01; Figure 5B). STAT1, pSTAT1, and pSTAT2 protein expression significantly also increased with increasing concentration of IFN-β (*P* < 0.01; Figure 5C).

## Discussion

The upper airway mucosa is continuously exposed to various microorganisms and respiratory viruses; respiratory viruses are frequently detected in the airway of patients with chronic inflammatory nasal diseases without acute viral infection of the upper respiratory tract (28, 29). IFN-I response—the main player in the innate immune response elicited against respiratory viruses—may be affected by inhaled stimulants, and is speculated to be involved in the pathogenesis of CRS. In this study, IFN-β, which is one of IFN-I, was overexpressed in type 2-skewed eosinophilic NP tissues in humans. Eosinophilic chronic sinonasal inflammation and type 2 immune response were not definitely induced in the absence of IFN-I signaling, and CCL11 production was enhanced by exogenous IFN-β stimulation in NP stromal cells.

In addition to the proven role of IFN-I in antiviral immunity, previous studies suggested the possible immune-modulatory function of IFN-I in the pathogenesis of CRS. Zhang et al. reported a higher number of IRF-7^+^ and IFN-β^+^ cells in the NP tissue from ECRS subjects as well as a positive correlation between IFN-β mRNA expression and the number of tissue eosinophils (13). Although they suggested the potential contribution of increased IFN-β level on mucosal eosinophilia, they did not present the causal relationship between IFN-I pathway and ECRS by using an appropriate ECRS animal model. We had previously found that respiratory virus-induced IFN-I response did not significantly affect the nasal epithelial cells from CRS patients (30). However, our study was targeted only at the nasal epithelial cells and did not discriminate between ECRS and non-ECRS cases. In the present study, we aimed to compensate the shortcomings of these previous studies, and therefore, investigated the role of IFN-I on the pathogenesis of CRS. Our results showed that IFN-β expression was higher in eosinophilic NP tissue and that IFN-β level positively correlated with IL-5, IL-13, and CCL11 levels as well as the number of eosinophils in the NP tissue and Lund-Mackay CT scores, which are hallmarks of eosinophilic CRSwNP (1–4, 26). Therefore, these data indicate the contribution of IFN-β to the pathogenesis of eosinophilic CRSwNP in terms of protein levels and clinical significance. One previous study reported that there were no significant differences in IFN-I gene expression in sinonasal tissues and IFN-I in nasal lavage fluid from CRSwNP patients compared to those from control and CRS without NP patients (31). However, because they determined the protein levels in nasal lavage fluid rather than sinonasal tissue, our findings, which showed an increase in IFN-I gene and protein expression in the sinonasal tissue, seem more reasonable.

Regarding the IFN-I^+^ cells, unlike the expression displayed mainly by epithelial cells and some immune cells, as suggested by the previous study (13), we found that IFN-β was expressed chiefly by the infiltrating cells in the lamina propria and some epithelial cells. IFN-I is predominantly produced by the DCs and macrophages after sensing pathogenic products (6). DCs are known to be significantly increased in the NP tissue from ECRS compared to that from the control and non-ECRS groups (32). In the present study, IFN-β^+^ cells in the lamina propria were highly co-localized with CD11c^+^ cells. CD11c is expressed not only on conventional DCs, but also on plasmacytoid DCs (pDCs), monocytes, and macrophages. Cells positive for BDCA2 (CD303), a marker exclusively expressed on human pDCs, were co-localized with approximately half of the IFN-β^+^ cell population (Figure E2). These results suggest that CD11c^+^ cells, including pDCs, might contribute to IFN- β production within the sinonasal mucosa, and are closely linked to ECRS development.

The involvement of IFN-I in eosinophilic inflammation was primarily investigated in association with acute respiratory viral infections in patients with asthma, a hallmark type 2 immune response-mediated respiratory disease, but not in patients with CRS. IFN-I response impairment leads to the exacerbation of asthma symptoms and eosinophilic inflammation in the lung (33–35). Some recent studies, therefore, studied IFN-I-mediated negative regulation of type 2 immune response after respiratory viral infection in *Ifnar1*^−/−^ mice (36, 37). In contrast to the known association between impaired IFN-I and increased eosinophilic inflammation, our study showed that IFN-β overexpression was associated with type 2-skewed eosinophilic NP tissue in humans. To further probe the role of IFN-I in chronic eosinophilic inflammation, we studied the cause-effect relationship between IFN-I response and type 2 skewed-chronic eosinophilic inflammation by using a relevant animal model. WT and *Ifnar1*^−/−^ mice were intranasally treated with AP + OVA for a long period to develop chronic eosinophilic sinonasal inflammation. We previously developed the ECRS model by direct inoculation of the fungal protease, which is known to be a human airway allergen and often used to induce allergic CRS and allergic asthma in mice (19, 20, 27, 38). We found that the chronic eosinophilic sinonasal inflammation and type 2 immune response, including the production of IL-4, IL-5, IL-13, and CCL11, were less severe in *Ifnar1*^−/−^ mice than in the WT mice. These results suggest that the IFN-I signaling pathway may play a role in development of ECRS. Moreover, IFN-I response during chronic upper airway inflammation may positively regulate type 2 immune response, in a different manner, with respect to its role in acute inflammation during respiratory viral infection wherein IFN-I response inhibit type 2 immune response (36, 37).

CCL11 was significantly increased in the eosinophilic NP tissue than in the NP tissues from non-eosinophilic groups, as well as was significantly increased in the WT AP + OVA challenged mice compared to the *Ifnar1*^−/−^ AP + OVA challenged mice. A positive correlation was noted between IFN-β and CCL11 expression in the NP tissue from ECRS patients. CCL11 induces eosinophil trafficking to the mucosa and its activation (39, 40), and plays a central role in the pathogenesis of eosinophilic NP (41). Thus, CCL11 is suspected to be an integral component involved in the effect of IFN-I on the development of ECRS. When NP stromal cells were exposed to exogenous IFN-β, CCL11 production was promoted by IFN-β in a dose-dependent manner, and not only STAT1 and STAT2 mRNA but also STAT1, pSTAT1, and pSTAT2 protein expression were also enhanced by IFN-β. STAT1 and STAT2 are key components of the transcription factor complex in IFN-I signaling pathways, and their activation induces IFN-stimulated gene expression (42). However, the mRNA expression of IRF9 forming an IFN-stimulated gene factor 3 complex with STAT1 and STAT2 was not changed by IFN-β stimulation. Further studies are required to understand whether STAT1, STAT2, and IRF9 are necessary for CCL11 production by IFN-β. Another possible explanation of the association between the upregulated IFN-I response and increased eosinophilic sinonasal inflammation is, although it was not proven in our study, IFN-I-induced overproduction of B cell-activating factor of the tumor necrosis factor family, leading to the local induction of IgA and activation of eosinophils in the NP (43–45).

The present study has some limitations. Respiratory viruses harbored in the tissue samples were not studied in human NP tissue. We omitted this experiment because a previous study found no significant difference in the virus detection rate of the epithelial cells from the control, non-ECRS, and ECRS groups (13), and therefore, we deemed the detection of viruses in the tissue homogenates from the control and NP tissues as unnecessary. Second, because of the relatively smaller sample, it is necessary that the findings be validated in a larger sample, to strengthen the reliability of our results. Third, we could not reveal the definite mechanism by which IFN-I response is linked with enhanced inflammation in ECRS, and the specific mechanism by which IFN-I promotes CCL11 production. Therefore, further studies are needed to clarify the exact role of IFN-I in the eosinophilic inflammation of the upper airway mucosa.

Despite limitations, our study offered novel findings that enhanced IFN-β response, contributes to chronic eosinophilic inflammation by increasing CCL11 production in the sinonasal mucosa. This result suggests that IFN-β may play a critical role in the development of ECRS, and therefore, may be a therapeutic target for ECRS.

## Author contributions

JHK designed the study, acquired and analyzed the data, and wrote the manuscript. YJJ analyzed the data and wrote the manuscript. JYL, SK, and YLL acquired the data. M-NK analyzed the data.

### Conflict of interest statement

The authors declare that the research was conducted in the absence of any commercial or financial relationships that could be construed as a potential conflict of interest.
