# Supplementary material for: Enhanced Interferon-β Response Contributes to Eosinophilic Chronic Rhinosinusitis
Source: Front Immunol. 2018 Oct 16;9:2330. doi: 10.3389/fimmu.2018.02330 (PMC6232691; doi:10.3389/fimmu.2018.02330)
Supplement: Supplementary file 1 [file Data_Sheet_1.pdf]

Online Supplementary Information

Enhanced interferon-β response contributes to eosinophilic chronic rhinosinusitis

Yong Ju Jang,[1] Ji Youn Lim,[1] Seoyeon Kim,[1] Yoo La Lee,[1] Mi-Na Kweon,[2] Ji Heui Kim[1*]

Corresponding author: Ji Heui Kim (jhkim0217@amc.seoul.kr)

Data S1. Supporting Methods

Real-time PCR for type I IFN in human tissue

Total RNA was extracted from 45 μL of tissue sample with the RNeasy mini kit (Qiagen GmbH, Hilden, Germany) according to the manufacturer's instructions. cDNA was synthesized using M-MuLV reverse transcriptase (RT-&GO^{TM} kit; MP Biomedicals, Santa Ana, CA) according to the manufacturer's protocol. Quantitative real-time PCR was performed using each cDNA with the Light-Cycler 480 SYBR Green I Master (Roche, Mannheim, Germany). PCR primer sets used for the detection of glyceraldehyde-3-phosphate dehydrogenase (GAPDH), *IFNA* and *IFNB* are listed in Table E1. The reaction mixture was prepared by the addition of a primer set to a LightCycler® real-time PCR system following the manufacturer's instructions. Reaction conditions were as follows: initial denaturation at 95°C for 3 min, followed by 40 cycles of denaturation at 95°C for 20 s, annealing at 55°C for 20 s, and extension at 72°C for 20 s. Melting temperature analysis was also performed (60–95°C at 0.5°C increments for reading the fluorescent signals). To analyze the data, we used LightCycler® 480 software, Version 1.5 (Roche). The results were normalized to GAPDH expression, and the relative gene expression was calculated using the comparative $2^{-\Delta\Delta CT}$ method.

Human tissue homogenates

Fresh tissue was weighed and homogenized with 1 mL of a mixture containing Protease Inhibitor Cocktail (Roche), 1 mM EDTA, 10 mM Tris-HCl, 0.05% sodium azide, and 1% Tween80 (Sigma-Aldrich, St Louis, MO) per 100 mg of tissue. The homogenates were

centrifuged at 11,000 *g* for 10 min at 4°C; the supernatant was collected and stored at −80°C until analysis.

Nasal polyp stromal cell culture

NP stromal cells were isolated from NP tissue as described previously (E1). In brief, NP tissue samples were washed with PBS containing 10,000 U/mL penicillin, 10,000 µg/mL streptomycin, and 25 µg/mL amphotericin B. Chopped tissues were plated in 100-mm culture dishes (Corning, Corning, NY), and cultured in Dulbecco's modified Eagle's medium/nutrient mixture F-12 (DMEM/F12; Gibco, Grand Island, NY), supplemented with 10% (v/v) fetal bovine serum (Gibco), at 37°C in fully humidified air containing 5% (v/v) CO_2. After purification of single NP stromal cells, the attached fibroblast-like cells were cultured for 7 days.

Western blot analysis

After lysing NP stromal cells, protein samples (15 µg/lane) were resolved by electrophoresis on 7.5% sodium dodecylsulfate (SDS)-polyacrylamide gels and then transferred to nitrocellulose membranes. Membranes were incubated in blocking buffer and then incubated with one or more of the following primary antibodies: anti-STAT1(catalog no. 9172; Cell Signalling, 1:1000), anti-phospho STAT1 (catalog no. 7649; Cell Signalling, 1:1000), anti-STAT2(Cell Signalling, 1:1000), anti-phospho STAT2 (catalog no. 07-224; Upstate, 1:500) and anti-β-actin(Santa cruz, 1:5000). After incubating with primary antibodies, membranes were washed and incubated with horseradish peroxidase-conjugated secondary antibodies (Santa cruz). Immunoreactive bands were visualized by enhanced chemiluminescence (PerkinElmer Life Sciences, Boston, MA) and normalized to relative to β-actin expression of

the same samples.

REFERENCES

E1. Kim JH, Kwon HJ, Jang YJ. Levocetirizine inhibits rhinovirus-induced up-regulation of fibrogenic and angiogenic factors in nasal polyp fibroblasts. *Am J Rhinol Allergy* (2011) 25:416-20.

Online Supplement Table and Figures

TABLE E1. Primers used for quantitative real-time PCR analysis

Primer	Sequence		Expected product size (bp)	Melting temperature (°C)
IFNA	S	GAAATACTTCCAAAGAATCAC	90	78.4
	A	GATCTCATGATTTCTGCTCT		
IFNB	S	AGCTGCAGCAGTTCCAGAAG	110	85.3
	A	GTCTCATTCCAGCCAGTGC		
IRF3	S	AGAGGCTCGTGATGGTCAAG	159	80.5
	A	GCAGGTAGGCCTTGTACTGG		
IRF7	S	TACCATCTACCTGGGCTTCG	146	90.4
	A	TGCTGCTATCCAGGGAAGAC		
IRF9	S	CCCGAAAACTCCGGAACTG	64	82.8
	A	CAGCACACTCCGGGAAACT		
STAT1	S	CTAGTGGAGTGGAAGCGGAG	252	79.0
	A	CACCACAAACGAGCTCTGAA		
STAT2	S	CAGGCTCATTGTGGTCTCTAAT	101	83.0
	A	GCCCTAGTTCCAGCTCTAATG		

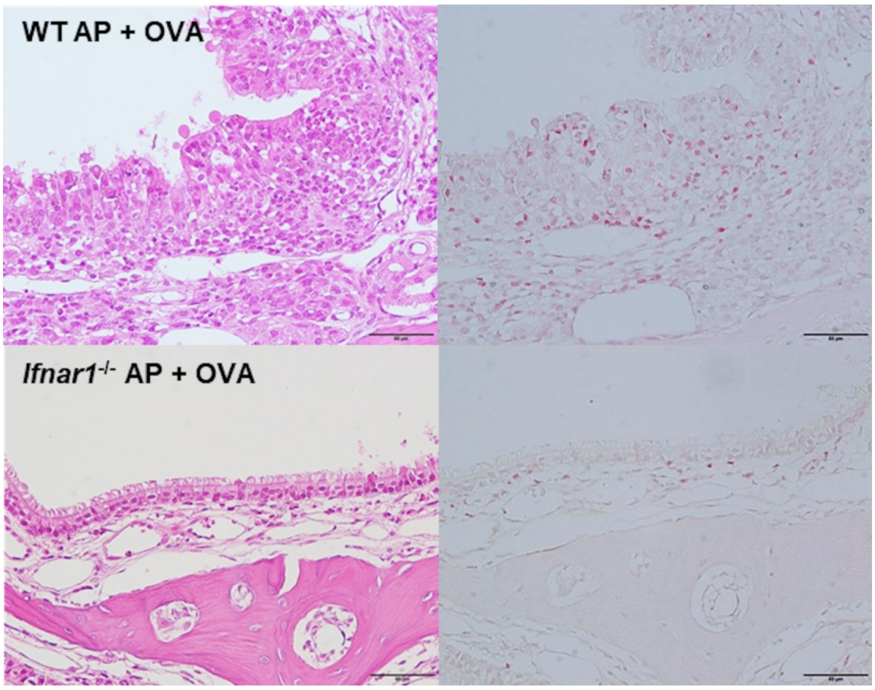

Figure E1. Polypoid lesions and severe eosinophilic inflammation developed in wild-type eosinophilic chronic rhinosinusitis mice intranasally treated with the protease from *Aspergillus oryzae* (AP) and ovalbumin (OVA) for 12 weeks, but this manifestation was not distinct in *Ifnar1*$^{-/-}$ AP and OVA challenged mice (magnification, ×400; scale bar, 50 μm).

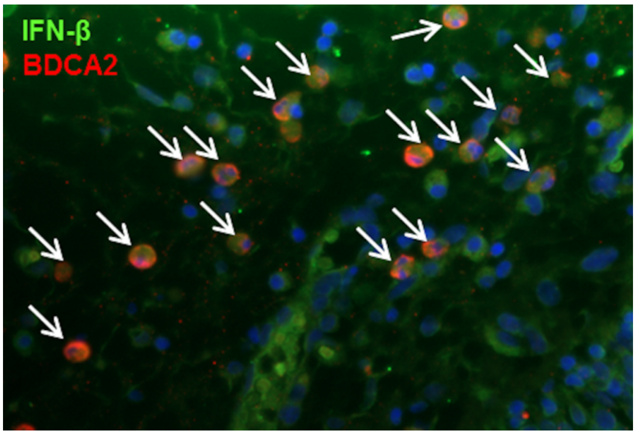

Figure E2. Immunofluorescence assay was performed with anti-IFN-β (green fluorescence), anti-BDCA2 (red fluorescence) for pDC, and DAPI (blue fluorescence) for nuclei (magnification, ×400). BDCA2+ cells (white arrow) were about 50% of IFN-β+ cells.
